# The Passing of a Fad

**Published:** 1902-12

**Authors:** 


### The Passing of a Fad.

FOR years the "healers" of the christian science cult have been treating the sick and propagating the theory that all illnesses were merely "beliefs;" they have made much money and have made the study of the published works of Mary Baker Eddy, the high priestess of the faith, a part of their treatments. The results have been profitable for the healers, who never treat for charity, and to Mrs. Eddy who is a millionaire several times over, according to the best evidence obtainable, as a result of the sale of her works.

Deaths due to neglect while under treatment at the hands of christian scientists began to occur and legal action was taken. For awhile there was not the slightest suspicion of conviction in any case. These cases of negligence, cases where pneumonia and diphtheria were treated by prayer, multiplied and the authorities awakened to a realisation of the fact that a serious problem confronted them. Being a mixture of nonsense and religion, christian science appealed chiefly to women with a hysterical tendency and to men with an eye to the main chance, hence the cult grew in numbers and many of the churches erected were marvels of gorgeous splendor. The christian scientists who took Mother Eddy's course of instruction at high rates for a couple of months' teaching and became "healers," went into practice, waxed rich and fat and sleek and prosperous. And now after many narrow escapes from imprisonment on the part of her "healers" Mother Eddy has reached out a protecting hand, and through force of circumstances has been compelled to issue an order which relegates christian science to the position which it should normally occupy—namely, a religious belief which is capable of doing much good in the world, and no harm to speak of. The edict which has been generally issued, and which it is believed is the result of the flagrant Quinby case in Westchester, reads as follows:

Mrs. Eddy advises that until the public thought becomes better acquainted with christian science, the christian scientists shall decline to doctor infectious or contagious diseases.

If the Eddyists do not admit the existence of disease, which heretofore has been heralded as their shibboleth, by what strange shifting of theory do they now claim the skill of diagnosis in infectious diseases, often difficult to the best trained physician?

In an interview regarding the edict a local healer stated that Mrs. Eddy's request did not in any way indicate that christian science was unable to cope with infectious or contagious disease, but was merely a tentative suggestion made to meet the exigencies of the present until the exact status of the christian scientists is defined by law.

This, then, is the beginning of the end of a fad which has numbered its victims by scores, for the law already defines the exact status of the christian science healer as an illegal practitioner of medicine.

In itself, and confined within legitimate limits, christian science is an order capable of much good. It has made better men and women of its followers; it has taught self-control and a broad-minded brotherly love which the churches of other religions might do well to emulate. True, they have their little differences, but they are never torn asunder by bitter dissension.

The only objection the JOURNAL and the medical profession at large has ever had to the scientists has been their belief in "beliefs" and their unauthorised and ignorant treatment of sick persons, especially children. The deaths of children while under christian science treatment for diseases which would have yielded to proper and intelligent medical management is the blackest stain on the history of Mrs. Eddy's organisation; it is a stain which will never be wiped out by any reform of methods on the part of the scientist church. "Suffer little children to come unto Me," was a divine command; but it was in nowise intended by Christ that the little children should come to Him suffering from neglect.

The church of Christ now takes its place where it belongs—among the religious orders of the world, and as such it is capable of doing much good, as it undoubtedly will; but so far as the practice of medicine is concerned the end is reached.

---

THE Maltine Company, as noted elsewhere, has made through the committee appointed to decide the matter, its award of prizes for the best essays on preventive medicine, in accordance with its offer of last winter. Both prizes, one of $1,000.00 and one of $500.00 go to residents of Philadelphia. The company deserves the congratulations of the medical profession for its liberality in thus stimulating literary work in the direction named, and scarcely could have chosen a more appropriate field for the manifestations of its generosity.

---

RECENT examinations of the city water supply indicate that it is contaminated with disease germs. It is difficult to fix the origin of the pollution. The health department has been making bacteriologic examinations and sufficient information is obtained to justify the recommendation that all drinking water should be boiled. It would be well also if it were filtered after boiling.

---

ONE of the Buffalo evening newspapers (?) just on the eve of the election made a despicable attempt to array the state medical society against Governor Odell. It published a statement that the society had unanimously adopted a resolution condemning the so-called lunacy bill of Governor Odell. It then published a list of the officers of the society and of the members residing in Erie County, and by innuendo strove to make it appear that the entire medical profession would oppose the Governor's reëlection. As a matter of fact the record of the society at its annual meeting, January, 1902, shows that Dr. Edward D. Fisher, of New York, offered the following resolution:

Protesting against the proposed lunacy bill, which removes all power from Boards of Managers and centralises it in the Lunacy Commission; believing that this would lower the medical standards of the State Hospitals. Seconded by Dr. E. B. Angell, and adopted unanimously.

The import of this is simply that those members of the state society then present, expressed themselves as opposed to such legislation. It did not mean, it could not mean, that a failure of the society's views to prevail would array that body against the Governor. It could not make republicans of democrats nor democrats of republicans; and no species of ratiocination however subtle or adroit, could make it so appear. The society is not a political body.

